# Polypoidal choroidal vasculopathy in a case with retinitis pigmentosa

**DOI:** 10.1007/s10792-012-9657-7

**Published:** 2012-11-07

**Authors:** Tomoka Ishida, Muka Moriyama, Kei Morohoshi, Yuu Furuse, Taiko Fukuda, Kyoko Ohno-Matsui

**Affiliations:** Department of Ophthalmology and Visual Science, Tokyo Medical and Dental University, 1-5-45 Yushima, Bunkyo-ku, Tokyo, 113-0034 Japan

**Keywords:** Polypoidal choroidal vasculopathy (PCV), Retinitis pigmentosa (RP), Ranibizumab, Indocyanine green angiography, Fluorescein angiography

## Abstract

There have been no reports describing polypoidal choroidal vasculopathy (PCV) in eyes with retinitis pigmentosa (RP). A 63-year-old woman who had been diagnosed as having RP was referred to us because of sudden onset of blurred vision in her right eye. Funduscopic examination revealed retinal findings typical of RP in both eyes. The macular area of the right fundus showed polypoidal lesions with massive hemorrhages. Fluorescein angiography and indocyanine green angiography showed multiple polypoidal lesions. Optical coherence tomography showed a large hemorrhagic retinal pigment epithelial (RPE) detachment and polypoidal lesions. The PCV subsided after three applications of anti-vascular endothelial growth factor (VEGF) therapy and a single application of photodynamic therapy, but “mottled lesions” with hyper- and hypofluorescence appeared temporal to the macula after disappearance of hemorrhage. We present a case of PCV in an eye with RP. Further studies are necessary to clarify whether anti-VEGF therapies could affect RPE status in eyes with RP.

## Background

Macular choroidal neovascularization (CNV) is a relatively rare complication of RP [1–3]. PubMed search with “CNV” and “retinitis pigmentosa (RP)” on 14 September 2012 extracted only three articles [1–3] reporting a case with classic CNV diagnosed angiographically. However, PubMed search with “polypoidal choroidal vasculopathy (PCV)” and “retinitis pigmentosa” on the same date did not extract any articles. We present a case with development of PCV in an eye with RP.

## Subject and observations

**Fig. 1:**
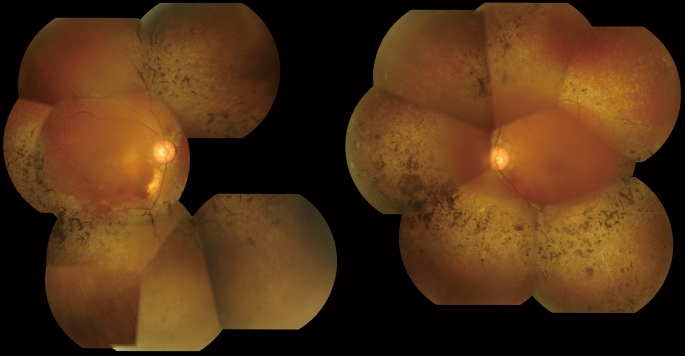
Color fundus photographs of both eyes showing findings typical of retinitis pigmentosa, such as bone spicule-like pigmentation, attenuated retinal vessels, and waxy pallor of optic disc

**Fig. 2:**
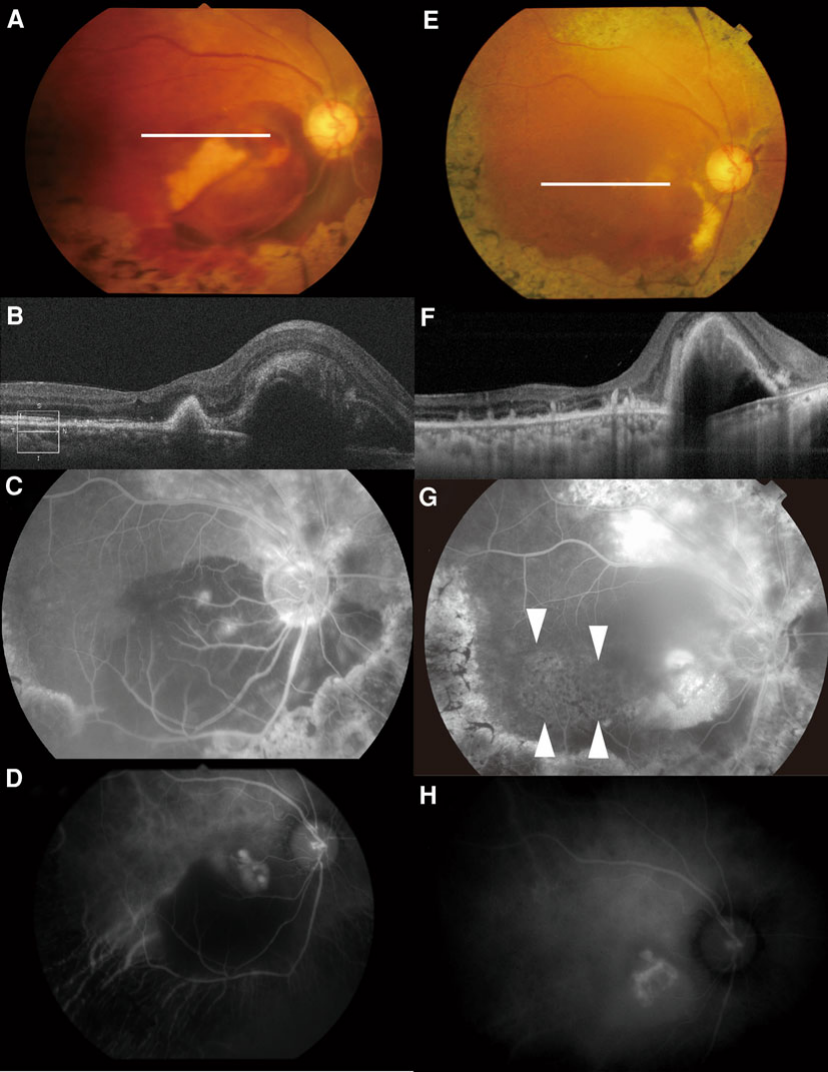
Retinal findings before (**a**–**d**) and after (**e**–**h**) intravitreous injection of ranibizumab (IVR) and photodynamic therapy (PDT). **a** Color fundus photograph of right fundus, showing the massive hemorrhage in the macula. **b** Optical coherence tomography (OCT) of right fundus, showing a large hemorrhagic retinal pigment epithelial detachment (PED) and polypoidal lesion. **c** Fluorescein angiography (FA) after 3 min of dye injection of right fundus, showing two points with dye leakage. **d** Indocyanine green angiography (IA) after 3 min of dye injection of right fundus, showing multiple polypoidal lesions. **e** Right fundus after treatment, showing the decrease of subretinal hemorrhage. **f** OCT after treatment, showing flat polypoidal lesions. **g** FA after 5 min of dye injection, showing mottled lesions with hyper- and hypofluorescence in the temporal region of macula (*arrowheads*). **h** IA after 10 min of dye injection of right fundus showing remnant polypoidal lesions with vascular networks

A 63-year-old woman presented with sudden onset of blurred vision in her right eye. She had been diagnosed as having retinitis pigmentosa (RP) without systemic complications. No members of her family had been diagnosed with RP. At initial examination, best-corrected visual acuity (BCVA) was 0.2 in the right eye and 1.0 in the left. Visual field examination using Goldmann perimetry showed concentric constriction of the field bilaterally and paracentral scotoma in the right eye. Funduscopic examination revealed retinal findings typical of RP, such as bone spicule-like pigmentation, attenuated retinal vessels, and waxy pallor of optic disc in both eyes (Fig. 1). The macular area of the right fundus showed polypoidal lesions between the optic disc and central fovea accompanied by massive hemorrhages. Fluorescein angiography (FA) showed two points with dye leakage, and indocyanine green angiography (IA) showed multiple polypoidal lesions (Fig. 2c, d). Optical coherence tomography (OCT) showed a large hemorrhagic retinal pigment epithelial detachment (PED) and polypoidal lesion (Fig. 2b).

After three consecutive intravitreal injections of 0.05 ml (0.5 mg) ranibizumab (IVR), a single application of photodynamic therapy (PDT) was performed. At 7 months after the last intravitreous ranibizumab injection and 2 months after PDT (Fig. 2e–h), the subretinal hemorrhage was decreased and polypoidal lesions became flat on OCT (Fig. 2f), although PED remained unchanged. Mottled lesions with hyper- and hypopigmentation appeared temporal to the macula after disappearance of hemorrhage (Fig. 2g). At the final visit, constriction of visual field had not progressed and BCVA was maintained during follow-up.

## Conclusions

Although the pathogenesis of PCV has not been fully clarified, PCV has been reported to occur in association with other retinal degenerative disorders such as tilted disc syndrome, high myopia, angioid streaks [4, 5], and central serous chorioretinopathy [6]. Martine et al. suggested the possibility that various factors such as impaired choroidal blood flow and degeneration of retinal pigmented epithelium could cause development of PCV [4]. In addition to RPE alterations, it has been reported that reduction of choroidal blood flow in clinical settings as well as histomorphological changes of choroidal blood vessels occur in eyes with RP [7–9].

The PCV in our patient subsided after three injections of ranibizumab and a single application of PDT. We performed PDT to obtain closure of polypoidal lesions. Although there have been no reports of PCV in RP, the two case reports [2, 3] describing classic CNV in RP found that the CNV in these two patients responded well to IVB.

Adverse effects of intravitreal injections of anti-vascular endothelial growth factor (VEGF) drugs in eyes with RP have not been reported, whether this therapy was to treat CNV [2, 3] or cystoid macular edema [10, 11]. In our patient, mottled fluorescence with a mixture of hypo- and hyperpigmentation appeared temporal to the macula after disappearance of hemorrhage. The RPE alterations observed in our patient appear similar to what was found to develop in four young females with classic CNV treated by PDT [12], and further studies are necessary to clarify whether such RPE alterations develop in association with RP.
